# Investigation of Elemental Mass Spectrometry in Pharmacology for Peptide Quantitation at Femtomolar Levels

**DOI:** 10.1371/journal.pone.0157943

**Published:** 2016-06-23

**Authors:** Emmanuelle Cordeau, Carine Arnaudguilhem, Brice Bouyssiere, Agnès Hagège, Jean Martinez, Gilles Subra, Sonia Cantel, Christine Enjalbal

**Affiliations:** 1 Institut des Biomolécules Max Mousseron (IBMM), UMR 5247, Université de Montpellier, CNRS, ENSCM, Place Eugène Bataillon, 34095 Montpellier cedex 5, France; 2 Laboratoire de Chimie Analytique Bio-inorganique et Environnement LCABIE-IPREM, UMR 5254, Hélioparc, 2 av. Pr. Angot, 64053 Pau, France; 3 Institute of Analytical Sciences (ISA), UMR 5280, CNRS, Université Lyon 1, 5 rue de la Doua, 69100 Villeurbanne, France; University of Helsinki, FINLAND

## Abstract

In the search of new robust and environmental-friendly analytical methods able to answer quantitative issues in pharmacology, we explore liquid chromatography (LC) associated with elemental mass spectrometry (ICP-MS) to monitor peptides in such complex biological matrices. The novelty is to use mass spectrometry to replace radiolabelling and radioactivity measurements, which represent up-to now the gold standard to measure organic compound concentrations in life science. As a proof of concept, we choose the vasopressin (AVP)/V1A receptor system for model pharmacological assays. The capacity of ICP-MS to provide highly sensitive quantitation of metallic and hetero elements, whatever the sample medium, prompted us to investigate this technique in combination with appropriate labelling of the peptide of interest. Selenium, that is scarcely present in biological media, was selected as a good compromise between ICP-MS response, covalent tagging ability using conventional sulfur chemistry and peptide detection specificity. Applying selenium monitoring by elemental mass spectrometry in pharmacology is challenging due to the very high salt content and organic material complexity of the samples that produces polyatomic aggregates and thus potentially mass interferences with selenium detection. Hyphenation with a chromatographic separation was found compulsory. Noteworthy, we aimed to develop a straightforward quantitative protocol that can be performed in any laboratory equipped with a standard macrobore LC-ICP-MS system, in order to avoid time-consuming sample treatment or special implementation of instrumental set-up, while allowing efficient suppression of all mass interferences to reach the targeted sensitivity. Significantly, a quantification limit of 57 ng Se L^-1^ (72 femtomoles of injected Se) was achieved, the samples issued from the pharmacological assays being directly introduced into the LC-ICP-MS system. The established method was successfully validated and applied to the measurement of the vasopressin ligand affinity for its V1A receptor through the determination of the dissociation constant (Kd) which was compared to the one recorded with conventional radioactivity assays.

## Introduction

Many issues in life science rely on the sensitive and specific concentration measurement of a biomarker present at trace levels in very complex biological matrices. Among all classes of biomolecules, peptides have gained in acceptance as potent drugs in the last few decades [1] and thus became a major topic of investigation in pharmacology. The standard quantitation method used in pharmacology implies the introduction of a radioactive element onto the targeted compound that is measured down to subnanomolar ranges [2]. Although very attractive performances are reached, this radiolabelling strategy suffers from severe drawbacks strongly limiting its applicability to laboratories authorized to use and store radioactive materials. In the search of alternative methodologies, fluorescent labelling has been successfully investigated for peptide quantitation [3]. However, anchoring a bulky fluorescent moiety to a biomarker could strongly affect its biological activity reducing the frame of interest of such approach. The inherent capabilities of mass spectrometry such as speed, sensitivity and specificity of detection makes this technique also particularly appropriate for targeting any molecules in a complex mixture. In contrast to molecular mass spectrometry that produces ions from entire organic entities, elemental mass spectrometry, known as inductively coupled plasma-mass spectrometry (ICP-MS), operates at very high temperatures (up to 8000 K) allowing to break all chemical bonds, the elements being converted into positively charged ions [4]. Among all elements of the periodic classification, metals exhibit very high ionization efficiency in ICP-MS making them particularly suited for elemental quantification in various domains [5]. The response of the metallic element is thus independent of the molecular environment allowing absolute quantification. This feature is of crucial importance to tackle concentration measurements of a highly diluted specific organic substrate in very complex biological samples. However, selecting the ICP-MS technology infers one major constraint on the experimental protocol: the targeted compound must contain at least one element that produces abundant ions in the ICP-MS ionization source. Although the potential of metallic tags in conjunction with ICP-MS analysis has been recognized to quantify peptides [6], the fact that metals were complexed to chelators (such as EDTA, DOPA, DPTA) anchored to the sequences [7] could potentially affect the peptides physico-chemical properties and their bioactivity. Thus, we preferred to investigate an analytical strategy based on the covalent binding of the ICP-MS element onto the studied molecule in order to minimize structural modification. Since recent developments broaden the scope of ICP-MS from metal to hetero elements analysis (S, P, Se, …) and of metal-containing or metal-tagged organic compounds and biomolecules [8], we investigated the insertion of a selenium atom in the organic compound of concern to act as a tag in elemental MS quantitative analyses. The selection of selenium was governed by the following reasons. First of all, this element, which is detectable by ICP-MS, is not commonly found in biomolecules and bioactive compounds. It means that it can be considered as a relevant quantification probe as demonstrated by Gammelgaard *et al*. to estimate the cellular up-take of a cell-penetrating peptide [9]. Secondly, in contrast to metals which required chelates to be associated with bioactive compounds, this element can be covalently bound to the targeted biomarker (peptide/small molecule) according to conventional organic chemistry procedures. Indeed, as member of chalcogen elements, sulfur (S) and selenium (Se) share chemical reactivity similarities [10], enabling the straightforward design of selenium-containing compounds by simply exchanging both atoms (sulfur replaced by selenium). Moreover, multiple incorporations of selenium were also envisaged to enhance the detection sensitivity. To be able to probe such behavior, peptides were chosen as model compounds since selenium labelling can be very easily introduced through the incorporation in the sequence of commercially available seleno-containing amino acids such as selenomethionine and selenocysteine (Sec) in place of methionine and cysteine (Cys) residues, respectively. We report in this paper the quantitation of a selenium-containing peptide bearing two Sec residues in the sequence by LC-ICP-MS. Although specific pieces of equipment relying either on capillary liquid chromatography [11] or desolvator system [12] have been described to optimize sensitivity, we aimed at using conventional macrobore liquid chromatography hyphenated with an ICP-MS instrument routinely used for analyses of organic matters [13]. Such instrumental configuration includes a cooled spray chamber and oxygen in the nebulizer gas. Method development is discussed and the performances of the investigated methodology (sensitivity, quantitation limits, accuracy, precision and interference) are evaluated for the specific targeted peptide monitoring. Finally, particular attention was devoted to the analyses of sample matrix handled in pharmacology. Results are provided from concentration figures acquired from cell cultures including the successful measurement of the dissociation constant (Kd) defining the affinity of the selenium labelled vasopressin peptide ([Se-Se]-AVP) for its V1A receptor which was verified by conventional radioactive measurements.

## Material and Methods

### Chemicals and safety considerations

All chemicals and solvents used for analyses were of analytical grade. All other chemicals were reagent grade or higher and were used as received unless otherwise specified. HF is a hazardous acid, which can result in serious tissue damage if burns were not appropriately treated. HF treatment used for peptide synthesis should be performed in a well-ventilated fume hood with appropriate face shield and double layered nitrile gloves.

### Synthesis of selenium-containing AVP peptide ([Se-Se]-AVP)

Peptide was prepared by solid phase peptide synthesis (SPPS). To avoid side reactions, like deselenation or racemization of the N-protected selenocysteine derivative occurring under basic conditions [14], the Boc strategy was preferred to the Fmoc strategy which requires iterative piperidine treatments for Fmoc deprotection steps. Synthesis was carried out using Boc in situ neutralization SPPS protocol as described by Alewood and co-workers [15]. Selenocystine as well as all Boc-protected amino acids except Boc-Sec(MeBzl)-OH were supplied by Iris Biotech GmbH (Germany). Boc-Sec(MeBzl)-OH was synthesized from commercial selenocystine according to a published protocol [16] and incorporated in the peptide sequence as a standard amino acid. Peptide was cleaved from the solid support with HF treatment. During this final cleavage step allowing side-chain deprotections together with peptide release in solution, the free selenol groups (Se-H) underwent prompt intramolecular oxidation leading to the diselenide bridge. Synthetic peptide was purified by preparative liquid chromatography (Waters Milford, CA), lyophilized and stored as a white powder. The overall yield of pure peptide was 74%. The experimental procedure is described in the supporting information (S1 Protocol).

### Quality control of the synthetic selenium-containing AVP peptide ([Se-Se]-AVP)

**Fig 1:**
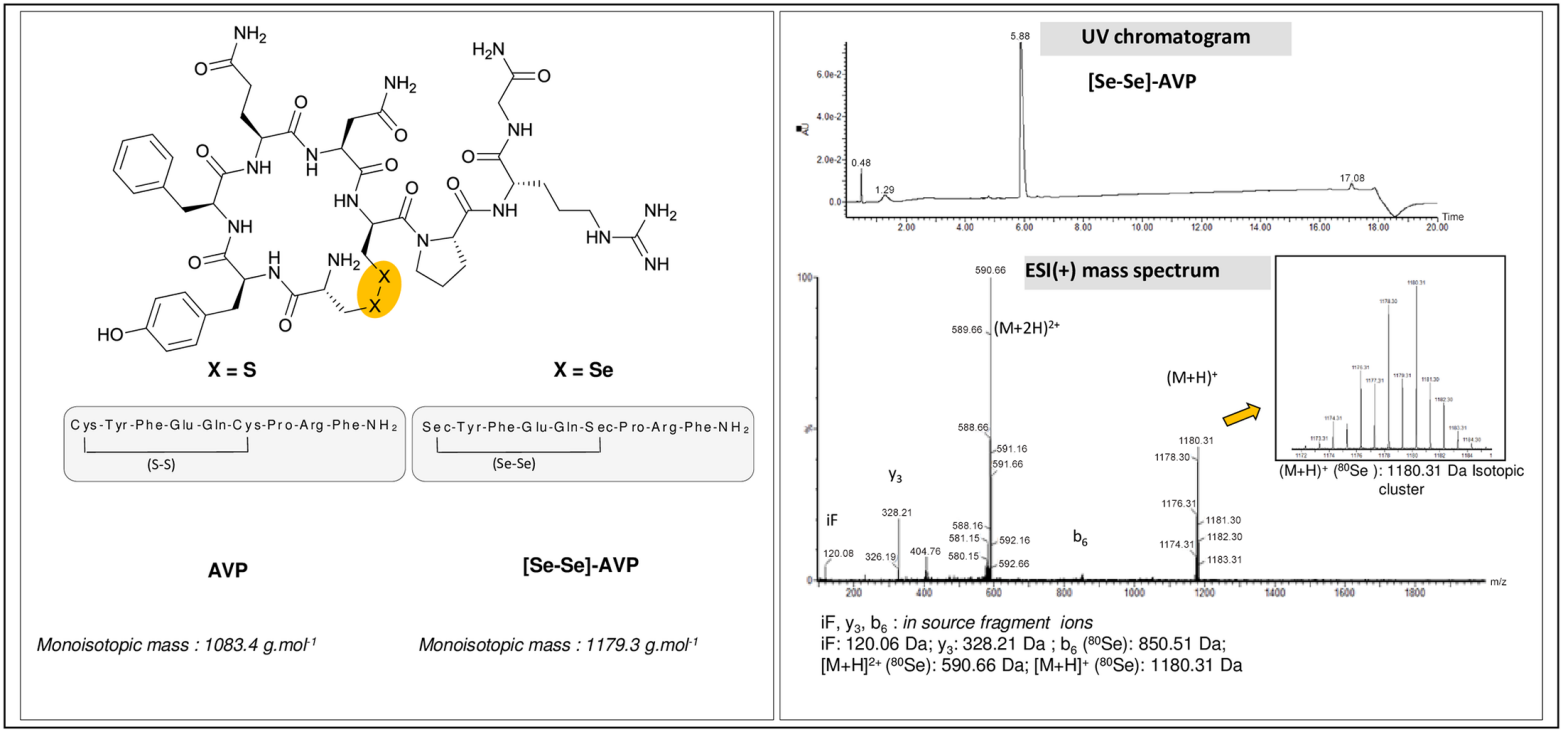
AVP and selenium labelled AVP.

Selenium possesses multiple stable isotopes that give raise to recognizable isotopic patterns in molecular mass spectrometry analyses performed at the ‘Plateforme Technologique Laboratoire de Mesures Physiques’ (IBMM, Université de Montpellier). Peptides were thus characterized by MALDI-Tof (Ultraflex III, Bruker) and ESI-QTof (Synapt G2S, Waters) techniques in order to properly check the values of the monoisotopic masses and the isotopic clusters of the protonated molecular ions. As displayed in Fig 1, the expected singly and doubly protonated ions were observed as well as fragment ions issued from prompt in-source dissociations (fragmentation of the pendant tripeptide chain Pro-Arg-Gly-NH_2_ leading to complementary y_3_ and b_6_ ions). Purity of the peptide was evaluated by integrating the UV chromatogram acquired in LC-ESI-MS experiments (97%). Furthermore, elemental analysis (Vario Micro Cube, Elementar) was used to determine the net peptide content (NPC), through the measurement of nitrogen mass ratio in the sample (78%). This information, which represents the organic fraction of the sample, allowed to estimate the relative amount of material contaminating the peptides (counter-ions, salts, residual solvents, water, …). The final purity was calculated by multiplying the UV signal integration with the NPC figure *i*.*e*. 76%. Data are given in S2 Protocol.

### RP-LC-ICP-MS methods

Standard solution of inorganic selenium was purchased from SCP Science (Courtaboeuf, France). Water was distilled and purified by a Milli-Q system (Millipore, Saint-Quentin en Yvelines, France). LC-ICP-MS analyses were performed on a Dionex Ultimate 3000 HPLC system, equipped with a degasser, a quaternary pump and an auto sampler. The column was an Eclipse XDB-C8, 3.5 μm, 2.1 x 100 mm. Analyses were carried out without the use of the column oven, with a flow rate of 400 μL min^-1^ and an injection volume of 100 μL. The quadrupole ICP-MS system was an Agilent 7700 Inductively Coupled Plasma Mass Spectrometer equipped with a Micromist nebulizer at 400 μL min^-1^, S lenses, Scott chamber operated at -5°C and a collision reaction cell with helium flow at 9.6 mL min^-1^. The sampler and skimmer cones were made of platinum. Two isotopes, ^78^Se and ^80^Se, were monitored with a dwell time of 960 msec/isotope.

### Biological matrices

Chinese Hamster Ovary (CHO) cells stably expressing the human V1A receptor, which have been suspended in phosphate buffer saline (PBS) buffer, were centrifuged. The buffer was removed, a 1% TFA solution was then added and [Se-Se]-AVP peptide solution was spiked at 75 ng Se kg^-1^. The monitored elements were ^44^Ca, ^58^Ni, ^60^Ni, ^64^Zn, ^66^Zn with dwell time of 200 msec/isotope and ^76^Se, ^77^Se, ^78^Se, ^80^Se, ^82^Se with dwell time of 300 msec/isotopes. Quantitation was performed by external calibration with [Se-Se]-AVP peptide and based on peak area measurement.

### Saturation experiment

Saturation experiments assays were performed using CHO cells stably expressing the human V1A receptor. The selenium labelled ligand [Se-Se]-AVP was used as ligand. Experiments were carried out in 2 mL of binding buffer at pH 7.4 (PBS with 1 mM of aqueous solution of CaCl_2_; 5 mM of aqueous solution of MgCl_2_; 0.1% of bovine serum albumin (BSA); 40 μg mL^-1^ of Bacitracine and 1 mM of PMSF), on CHO cells previously sowed on 6 multi-wells plate (1000000 cells/well). Total binding was determined with increasing concentrations of [Se-Se]-AVP (0.05 nM to 80 nM) while for non-specific binding the same experiment was performed in the presence of a 2000-fold excess of AVP. Incubation was performed for 4 h at 4°C. Assays were performed in triplicate. After incubation, cells were washed three times with binding buffer and then dissociation buffer (1% trifluoroacetic acid (TFA) aqueous solution (v/v)) was added. Mixture was transferred in Eppendorf tube and centrifuged at 14500 rpm. Supernatant was directly analyzed by RP-LC-ICP-MS with the above described method. Data were treated with non-linear model fitting programs (GraphPad PRISM 4).

## Results and Discussions

### Selenium properties

Selenium, like oxygen, sulfur and tellurium, belongs to the chalcogen chemical group and thus share similar properties [17]. Oxygen and sulfur are found in many functional groups of amino acid side-chains and therefore are considered as fundamental constituents of peptides and proteins. To a lesser extent, selenium has also been found in few native proteins in the form of two natural amino acids *i*.*e*. selenocysteine (Sec) and selenomethionine (SeMet) [18,19]. The former is even considered as the 21^st^ proteinogenic amino acid, due to its role in selenium bioincorporation. Both residues are commercially available. That is why synthetic strategies based on the substitution of a proteinogenic sulfur-containing amino acid (cysteine or methionine) with its selenium analogue (selenocysteine or selenomethionine) are readily accessible. For instance, Gammelgaard and collaborators introduced a selenomethionine in place of methionine to follow by ICP-MS the cellular uptake of such modified peptide [9]. In another context, substitution of cysteine with selenocyteine in peptide is widely used, most notably by Alewood and co-workers [20] mainly to replace disulfide bridge by a diselenide bond engineered to better control the oxidative peptide cyclization and thereafter improve its proteolytic stability. We decided to follow such a well-documented efficient synthetic strategy to prepare selenocysteine-containing peptides. Obviously, the oxidative reactivity of free selenol group (Se-H) linked to the redox potential of selenium induces prompt dimerization through the formation of diselenide bridge [14] and hence hampers the substitution in a peptide sequence of a single cysteine residue by a selenocysteine. The scope of the presented selenium labelling methodology is thus restricted to methionine- or disulphide bridge-containing peptides.

### Design of a selenium-containing peptide

Vasopressin (AVP), an endogenous cyclic peptide acting as an antidiuretic hormone, possesses a disulfide bridge (S-S bond) that can be swapped to a diselenide linkage (Se-Se bond). Such chalcogen replacement should not affect its receptor affinity as demonstrated by Alewood and co-workers on similar peptides [20]. These authors and others have checked that the incorporation of Se instead of S was not causing steric changes in peptide folding [17]. Similar receptor affinities were measured for both sulfur- and selenium-containing sequences [20]. Moreover, the same result was obtained for proteins where the replacement of sulfur by selenium did not perturb their structures [21]. In the same manner, the incorporation of selenium was not found deleterious to maintain protein catalytic activity [22], even in the case where cysteine is directly engaged in the catalytic process as for cytochromes P450 [23]. The selenium labelled analogue ([Se-Se]-AVP) was synthesized using conventional solid phase peptide synthesis (SPPS) by replacing the two cysteine residues with two selenocysteines. The structures of AVP and selenium labelled AVP peptides are displayed in Fig 1. As expected, sulfur substitution by selenium atoms did not affect the affinity of the prepared selenium-containing vasopressin analogue for the V1A receptor. The affinity constant (Ki) of AVP and [Se-Se]-AVP were measured through conventional binding experiments with radiolabelled reference and were found similar at 3.79±1.32 nM and 0.75±0.24 nM, respectively (means of 2 independent experiments performed with 4 and 2 replicates, respectively, given with standard deviations (±SD)). The pharmacological protocols are detailed in S3 Protocol.

### Interferences for selenium quantification

Mass interferences stand for the main limitation for consistent elemental measurement of very low concentration in complex matrices [24]. Two kinds of interferences can be distinguished: the spectroscopic ones (known as isobaric and polyatomic interferences) that are principally induced by plasma gas and matrix component; and the non-spectroscopic ones (principally induced by matrix effects) which impact all instrumentation steps from sample introduction to detection. The major goal of the analytical method designed to quantify very low selenium concentrations was thus to find instrumental conditions suppressing all potential spectroscopic interferences (S1 Table) that can be encountered in the analyses of biological matrices. Selenium is particularly impacted by polyatomic interferences, especially the most abundant isotope of selenium (^80^Se: 49.61%) is interfered by the ^40^Ar_2_ aggregate formed in the ICP plasma. In order to avoid this bias, two instrumental set-ups were chosen. On the one hand, the ICP-MS was fitted with a collision reaction cell that was filled with helium gas aiming to break polyatomic aggregates. Indeed, among all different technologies developed to minimize polyatomic interferences, the collision/reaction cell technology showed good capabilities to decrease them [25] as it has been demonstrated by Guérin *et al*. [26] who improved by approximately a factor 10 the limit of quantitation for selenium present in foodstuffs for animals. On the second hand, coupling the ICP-MS with a separation technique such as liquid chromatography was recommended to avoid the simultaneous detection of selenium isotopes issued from the targeted peptide alongside the above mentioned interferences. The biological samples will definitely be contaminated by many salts which should not be retained on a reversed-phase stationary phase whereas the less polar selenium-containing AVP peptides should elute afterward under a higher percentage of organic content. Since this factor has a great impact on each ICP-MS step (aerosol generation, atomization/excitation/ionization processes, plasma properties and tolerances, interferences…) [27] and due to the difficulty to predict the chromatographic behavior of the studied peptide, the LC-ICP-MS method was carefully investigated as detailed below, in order to avoid deleterious non-spectroscopic interferences. In addition to ^80^Se monitoring, the isotope 78 was also considered for selenium quantitation in this study, being rather less abundant (^78^Se: 23.77%) but far less affected by ^40^Ar_2_ polyatomic interferences.

### LC-ICP-MS method development

Knowing that the [Se-Se]-AVP peptide was recovered from the pharmacological experiments in an acidic dissociation buffer (1% of trifluoroacetic acid (TFA) in water (v/v)), we chose to conduct all ICP-MS analyses for method optimization with samples diluted in such a solution. The injected samples were accordingly constituted by standard 1% TFA solutions spiked with either [Se-Se]-AVP or inorganic selenium. We investigated first different inlet modes available with the ICP-MS ionization source *i*.*e*. in Flow Injection Analysis (FIA) by means of the direct injection of sample solutions into the ICP-MS without any chromatographic separation and in actual LC-ICP-MS coupling mode, the separation module being equipped with a chromatographic reversed-phase stationary phase suitable for peptide separation. The elution conditions were evaluated in FIA and LC coupling by varying the organic solvent, the percentage of the acidic additive, the type of the reversed-phase stationary phase and the gradient tables. As previously discussed, both isotopes ^78^Se and ^80^Se were selected for quantitation. Furthermore, the isotope ^77^Se was also monitored to evaluate potential interferences on both targeted isotopes by comparing experimental isotopes ratio to theoretical ones. Method development was thus conducted with the simultaneous detection of selenium isotopes 77, 78 and 80 with a dwell time of 300 msec/isotope. Having established the best analytical protocol, only two isotopes, ^78^Se and ^80^Se, were then simultaneously monitored for quantitation purposes (LOQ determination with a dwell time of 960 msec/isotope). All results that were obtained for ^78^Se quantitation under different instrumental conditions used for optimization are discussed below. The second set of data recorded for ^80^Se is supplied in S4 Protocol.

### Injection mode: FIA-ICP-MS vs RP-LC-ICP-MS

**Fig 2:**
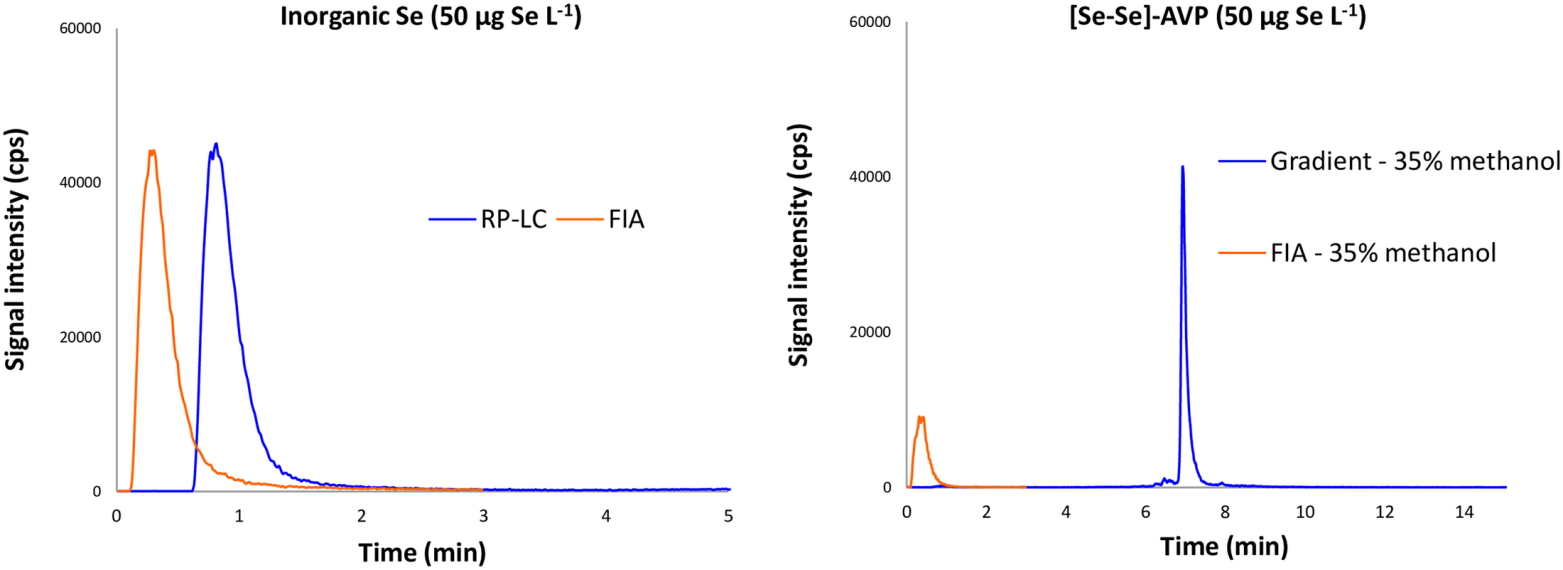
FIA *vs* RP-LC-ICP-MS analyses of [Se-Se]-AVP and inorganic Se.

An aqueous 1% trifluoroacetic acid (TFA) solution (v/v) of [Se-Se]-AVP at a concentration of 50 μg Se L^-1^ was analyzed in FIA-ICP-MS and with reversed-phase liquid chromatography (RP-LC-ICP-MS). Preliminary investigations on various reversed-phase stationary phase (data not shown) prompted us to choose a C8 reversed-phase stationary phase in combination with standard elution gradient profile (H_2_O + 0.1% formic acid and methanol + 0.1% formic acid, flow rate of 400 μL min^-1^ at 11% min^-1^ of methanol). Carbon content in specific proportion could enhance the signal detection [28,29], therefore in order to properly compare the FIA and RP-LC-ICP-MS injection modes on the signal detection, FIA acquisitions were performed with the organic percentage at which the peptide was eluted in RP-LC-ICP-MS run (*i*.*e*. 35% of methanol). Chromatographic separation provided a peptide signal that was almost 4 times more intense than the one recorded under FIA condition. The two overlaid chromatograms are displayed in Fig 2. Besides, the same set of experiments was conducted with inorganic selenium. A 1% TFA aqueous solution (v/v) supplemented with inorganic selenium at the same concentration (50 μg Se L^-1^) was analyzed with the two same modes of injection. As expected, inorganic selenium was eluted in RP-LC-ICP-MS in the solvent front at 100% water, so the FIA injection was carried out in the same solvent condition as shown in Fig 2. Separation by liquid chromatography had a huge influence on the peptide detection. The column recovery, estimated by the isotope 78 signal area detected from an LC-MS injection compared to the one measured in FIA analysis was evaluated at 183%. Since this phenomenon was not observed with inorganic selenium, which is not retained on the column, the stationary phase should limit diffusion occurring in FIA injection and provide some sample pre-concentration. The FIA injection mode was thus not further investigated for quantifying selenium-containing peptide. Most of the analytical development was conducted with the RP-LC-ICP-MS hyphenated technique.

#### Elution conditions: organic solvent

Proportion of organic solvent strongly affects plasma ionization yield and thus signal intensity [27]. The detection behaviour varies according to the nature of the organic compound but also of its relative content in a non-linear relationship, low amounts of organics causing signal enhancement whereas high concentrations induced decreased sensitivity [11]. Methanol and acetonitrile are the most often used organic solvents in reversed-phase liquid chromatography. We compared the signal intensity obtained for both of them by injecting the [Se-Se]-AVP sample (50 μg Se L^-1^) in water containing 1% of TFA (v/v) according to the previously used RP-LC conditions (C8 column, gradient elution at 400 μL min^-1^ from 0 to 100% of the organic solvent at 11% min^-1^), both mobile phases being supplemented with an organic modifier (0.1% of formic acid). Methanol afforded a 4-time more intense signal than acetonitrile. Despite the fact that the former is the prevalent organic solvent in conventional hyphenated molecular mass spectrometry technologies (LC-ESI-MS), the latter was thus chosen for the rest of the study.

#### Elution conditions: Gradient tables

**Fig 3:**
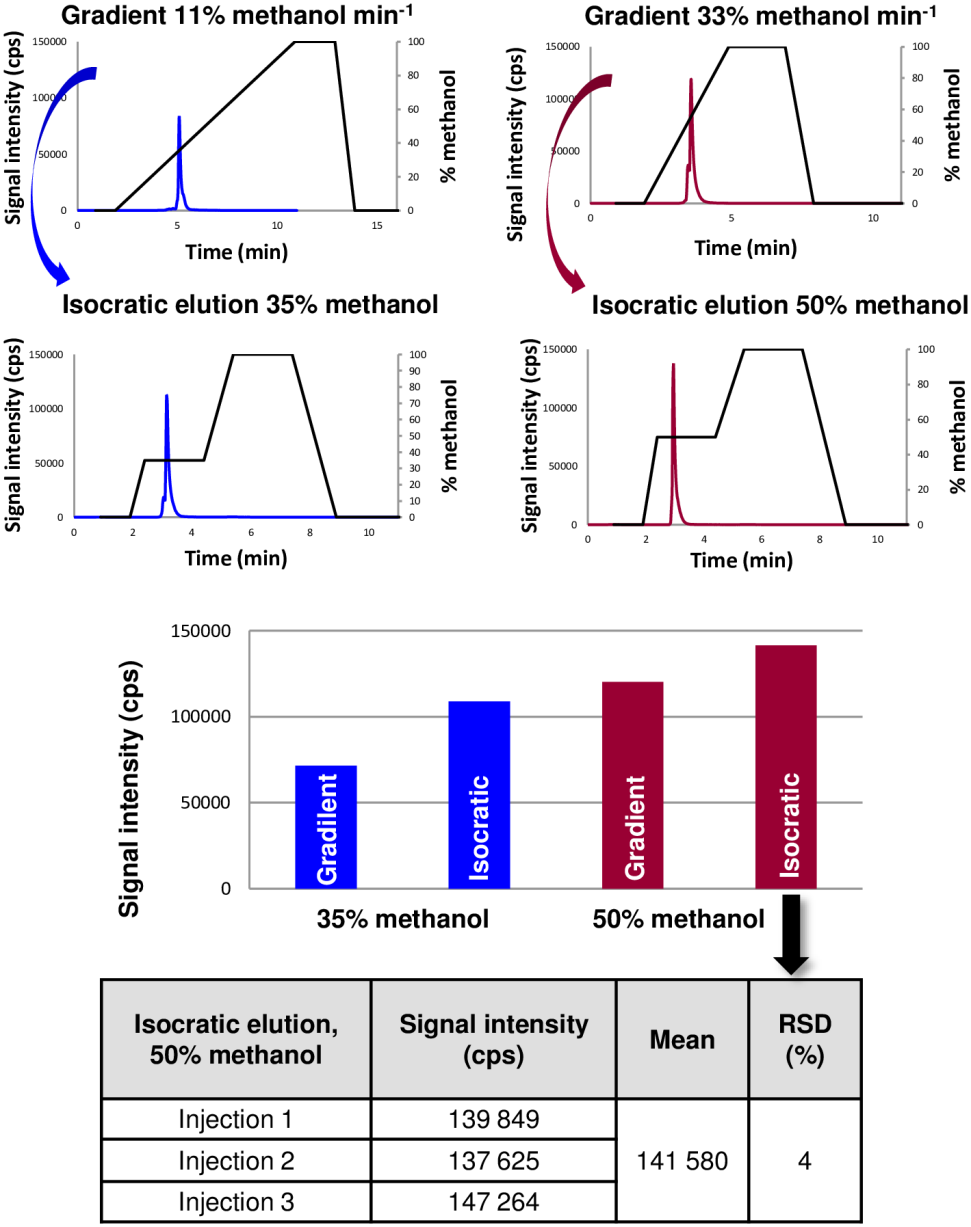
Elution profiles in RP-LC-ICP-MS analyses of [Se-Se]-AVP with the monitoring of ^78^Se.

Plasma tolerance (maximum amount of solvent that can reach the plasma per time unit) and robustness (plasma ability to accept matrix modifications without changes of its fundamental properties) are the two criteria allowing the evaluation of organic solvent effect [27]. To probe this point, an aqueous acidic methanolic solution of [Se-Se]-AVP at 31.3 nM (corresponding to 5 μg of Se L^-1^ in aqueous methanol (35%, v/v) supplemented with 1% of formic acid (v/v)) was injected to properly tune the ICP-MS parameters with the previously determined optimum organic content. Plasma power and sample gas flow rate were respectively increased from 1550W to 1600W and decreased from 0.76 L min^-1^ to 0.66 L min^-1^ affording approximately a two-fold signal intensity increase. Keeping all other RP-LC elution parameters unchanged, two different slopes of gradient elution were investigated to evaluate their impact on plasma tolerance and robustness and therefore on the signal intensity. Slow and fast elution conditions were applied with an increase of the organic solvent at 11% and 33% of methanol per minute, respectively. The [Se-Se]-AVP peptide sample was eluted at 35% and 52% of the organic solvent, respectively. The signal intensity was greater with the elution at 52% of methanol, the overall analysis time was quicker while keeping a valuable retention time to ensure efficient component separation (Fig 3). Isocratic elution modes were also evaluated. Indeed, better plasma stabilization, and thus a subsequent more intense ion signal and superior repeatability between consecutive injections should be obtained at fixed organic solvent percentage compared to rapidly changing content under gradient elution. In that condition, the so-called isocratic elution can also be considered as a step gradient. Signal intensity was compared for the two previously discussed percentages of methanol (35% and 50%) steadily introduced into the ICP source under isocratic elution. The results are shown in Fig 3. Isocratic elution with 50% of methanol provided the best signal (sharp peak and high intensity) at a retention time of 2.96 min. Three injections carried out in the row under this condition showed a relative standard deviation (RSD) of 4%. Despite such a good accurate and sensitive detection, potent interferences were also questioned. Bias between experimental and theoretical isotopic ratios was evaluated on the measured area with the simultaneous monitoring of isotopes 77, 78 and 80 as displayed in Table 1 under isocratic elution with 50% of methanol. Although the ^77^Se/^78^Se experimental ratio exhibited a lower bias than the ^77^Se/^80^Se one, the measurements were in the range of the expected values. Both ^78^Se and ^80^Se isotopes were not significantly affected at this concentration by polyatomic interferences in the blank dissociation buffer (1% aqueous TFA) and were thus both suitable for quantitation.

**Table 1:** Isotopic ratios measured with the isocratic elution at 50% of methanol.

	Mean of 3 injections				
	^77^Se	^78^Se	^80^Se	^77^Se/^78^Se	^77^Se/^80^Se
Area	152 062	500 560	1 166 791	30%	13%
RSD	1.4%	1.3%	1.3%		
Theoretical isotopic ratio				32%	16%
Bias				6%	23%

#### Elution conditions: Formic acid content in mobile phase

An organic acid additive is commonly used in the mobile phases of reversed-phase liquid chromatography. Thus, formic acid (FA) added in both water and methanol was introduced at two percentages (0.1% and 1%). The analyses were conducted from the previous aqueous 1% TFA peptide solution that has been diluted by a factor 10 (final concentration of 500 ng Se L^-1^). The [Se-Se]-AVP signal showed a detection sensitivity increased by approximately a 1.5 factor with 1% of FA compared to 0.1% in mobile phases. Furthermore, at this low concentration, chromatograms of both isotopes shown that isotope 80 was subject to some background noise whereas isotope 78 was not interfered as displayed in S4 Protocol.

#### Peptide adsorption/release and cross-contamination issue

**Fig 4:**
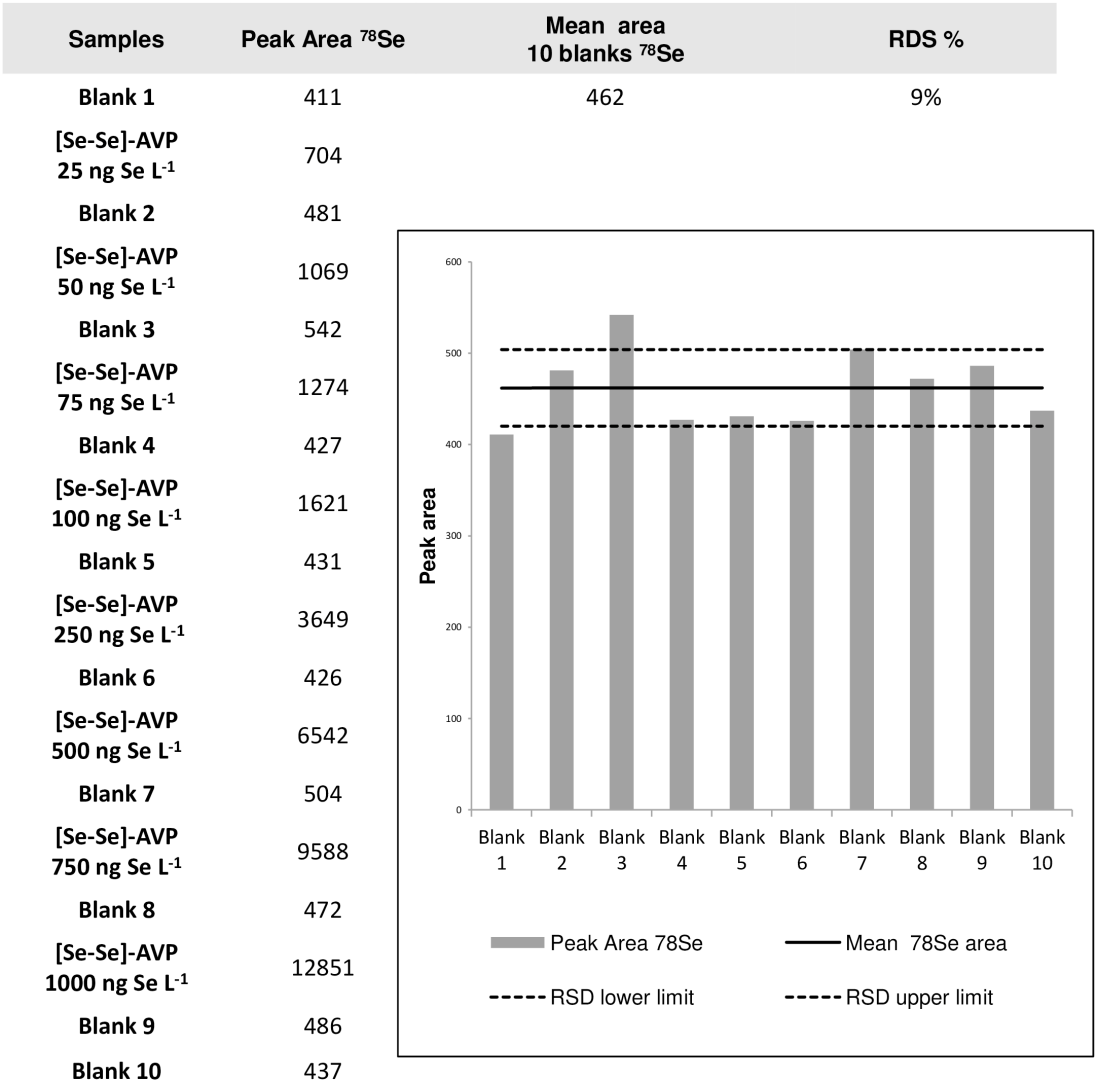
Injection of ten blanks with intercalated [Se-Se]-AVP peptide solutions at increasing concentrations.

In order to question the possible adsorption of the studied selenium-containing AVP peptide onto chromatographic system (injection needle, plastic/metal tubing, stationary phase, …), experiments were designed to evaluate the cleanliness of blank samples. If the peptide was progressively desorbed during the analytical series, a signal would be detected for blank samples. Contamination might be more potent in the case of highly concentrated samples. For this reason, we analyzed a set constituted by ten blank samples with intercalated injections of [Se-Se]-AVP peptide solutions from concentrations of 25 ng Se L^-1^ to 1000 ng Se L^-1^. No significant increase tendency was observed for the analyses of the 10 blank samples. Indeed, a tiny but steady signal was always found at the retention time of the peptide of interest, whatever its injected concentration. From these multiple measurements, we can conclude that this very small constant area certainly originated from contaminants that co-elute with the peptide at the beginning of the gradient stage. The relative standard deviation (RSD) of 9% (Fig 4) demonstrated no detectable peptide adsorption and release under the chosen RP-LC-ICP-MS conditions. We were then confident that no cross-contamination between injected samples will be occurring during the next analytical stages *i*.*e*. establishing the calibration curve and the quantitation method.

### [Se-Se]-AVP quantification by RP-LC-ICP-MS

#### Chromatographic protocol

The limits of detection (LOD) and quantification (LOQ) were evaluated with the best analytical conditions previously determined: Eclipse XDB-C8 column (3.5 μm, 2.1 x 100 mm), flow rate of 400 μL min^-1^, injection volume of 100 μL, solvent A: water with 1% of formic acid (v/v) and solvent B: methanol with 1% of formic acid (v/v). The elution profile included an isocratic step in pure water (100% solvent A) for 1 minute in order to elute all polar contaminants (mainly salts and very polar organic compounds) followed by a second isocratic stage at 50% of methanol during 2 minutes to isolate the expected selenium-containing AVP peptide (retention time of 2.96 min), and by a gradient to reach 100% of solvent B in 1 minute to separate any organic compound present in the sample. Finally, a cleaning step was applied for 2 minutes at 100% of methanol, then 100% of solvent A (starting elution conditions) were reached within 1.5 minutes, an extra 3 minutes of column conditioning gave a total run time of 11 minutes.

#### Calibration curve

**Fig 5:**
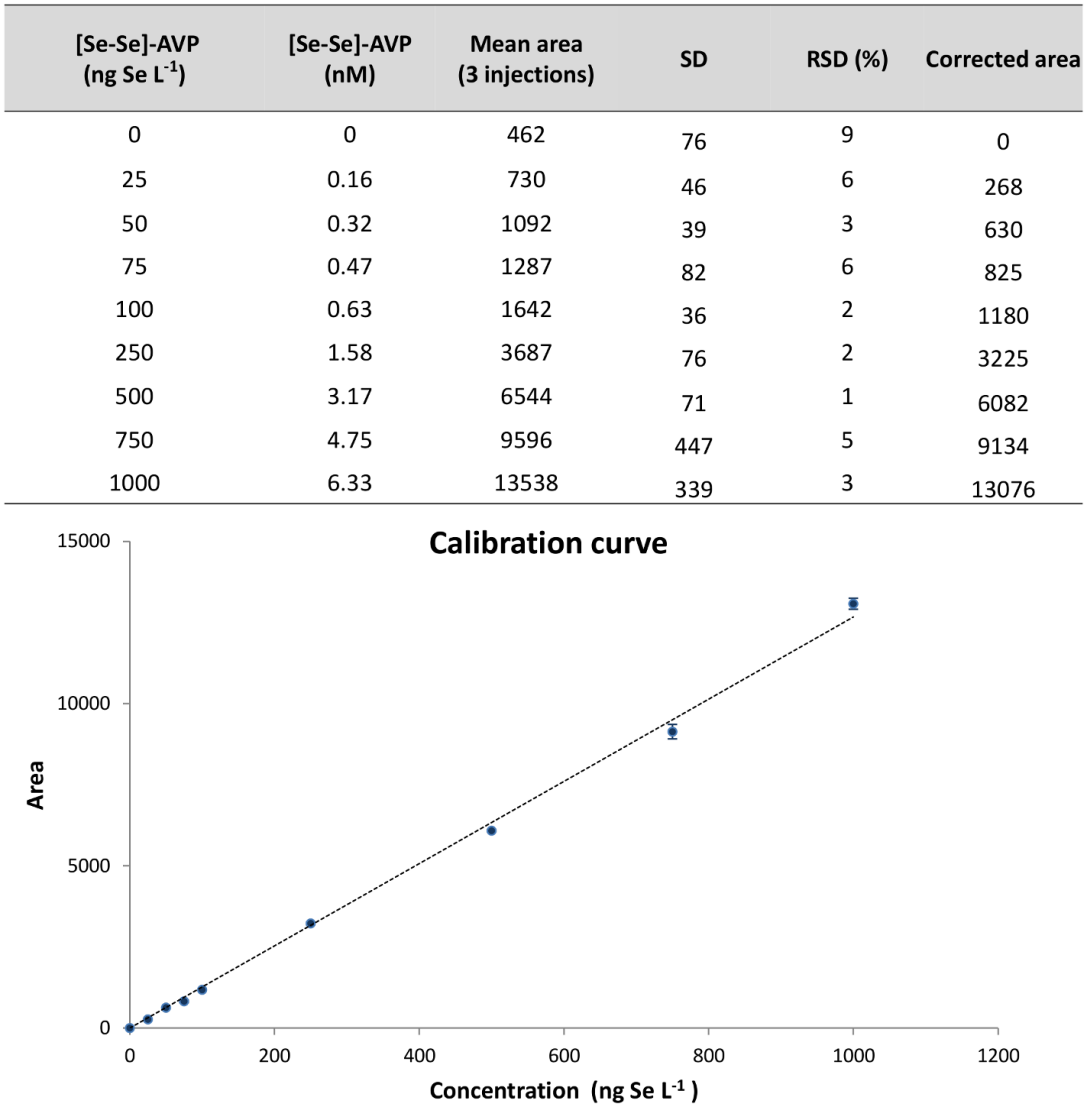
Calibration curve with ^78^Se monitoring.

A calibration curve has been performed with 8 levels of concentration (from 25 ng Se L^-1^ to 1000 ng Se L^-1^ of selenium corresponding to 0.16 nM to 6.3 nM of [Se-Se]-AVP peptide). The curve was found linear on the investigated dynamic range (R^2^ = 0.9991) as shown in Fig 5. Precision estimated from three repeated injections was lower than 6.4% (RSD) for each concentration, except for the blank sample which exhibited a non-null response with a RSD of 9% as previously described on ten injections. This repeatable selenium detection background deduced from blank samples was thereafter subtracted of analyzed sample areas providing corrected values that were taken into account for the calibration curve. According to the calculations below, the LOD and LOQ were estimated from the mean values of 3 calibration curves to be 17 ng Se L^-1^ (22 femtomoles of injected Se) and 57 ng Se L^-1^ (72 femtomoles of injected Se), respectively:

$$\text{LOD}\;=\;\frac{3\delta \left(b\right)}{a}$$

$$\text{LOQ}\;=\;\frac{10\delta \left(b\right)}{a}$$

with a representing the slope and δ(b) the standard deviation estimated on 10 blanks. As a matter of fact, an LOD of 400 picomoles of injected Se was depicted for selenium-containing peptide quantitation using macrobore RP-LC-ICP-MS instrument equipped with a membrane desolvator system [9]. The described experimental set-up and methodology proved to be more sensitive with an LOD of about 20 femtomoles of injected Se. Obviously, the use of nanoLC, which required a specific interface (microflow total consumption nebulizer) to be hyphenated to the ICP-MS instrument, allowed diminishing the LOD to around 0.5 femtomoles of injected Se [30].

Finally, the accuracy of the method have been evaluated on 3 levels of concentration including the LOQ. Percentages of error are lower than 9% (Table 2). The method was thus found suitable to measure the selenium content from biological matrices. We can note that bias observed at the LOQ concentration for isotope 78 (8.6%) was lower than the one observed for isotope 80 (18.6%) as displayed in S5 Protocol. This confirmed that, at lowest measured peptide concentration, the isotope 80 of selenium was slightly more interfered than the isotope 78.

**Table 2:** Method accuracy.

Injected concentration (ng Se L^-1)^	Calculated concentration from calibration curve (ng Se L^-1^)	Bias (%)
50	46	8.6
250	253	1.0
1000	1025	2.5

### Quantification in biological matricesBiological matrix spiked with [Se-Se]-AVP

**Fig 6:**
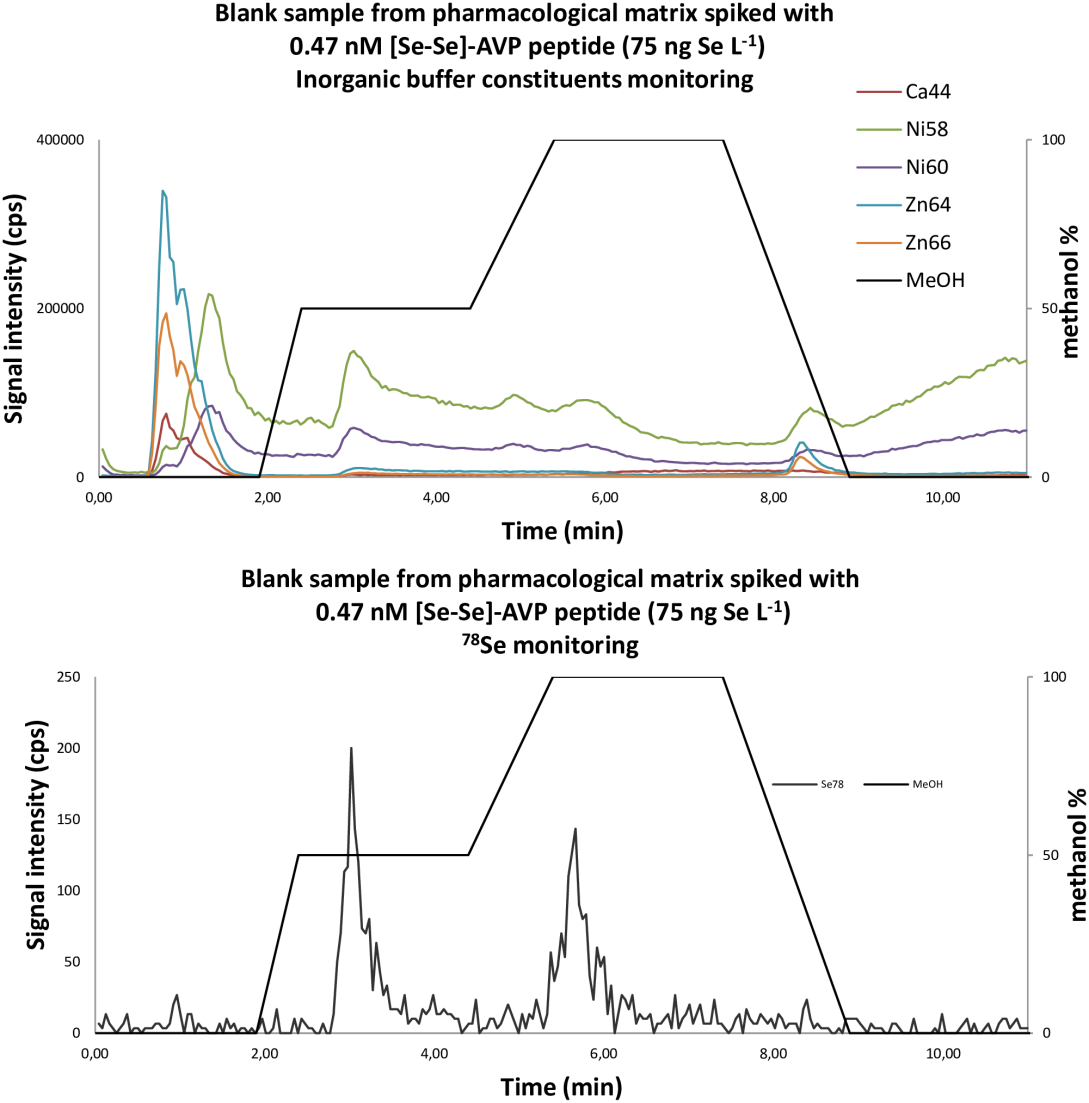
Overlaid RP-LC-ICP-MS chromatograms of a biological matrix spiked with [Se-Se]-AVP.

Quantitation was established by monitoring simultaneously both ^78^Se and ^80^Se isotopes together with the 3 other isotopes (^76^Se, ^77^Se, ^82^Se) for further proof of selenium detection. Buffers used in pharmacological experiments contain several constituents (such as Ca^2+^, Ni^2+^, Zn^2+^, Cl^-^…) that could interfere with this selenium isotope detection as stated in S6 Protocol. A biological medium (CHO cells suspended in PBS buffer) used commonly in pharmacological experiments in the laboratory was spiked with [Se-Se]-AVP at a concentration of 0.47 nM (75 ng Se L^-1^) and analyzed with the previously optimized chromatographic procedure. Elements that can most probably interfere with both selenium 78 and 80 isotopes (especially Ca, Ni and Zn) were monitored. As seen in Fig 6, the isocratic elution at 100% of water during 1 minute at the beginning of the elution profile allowed discarding the majority of these metals. The quantitation based on the ^78^Se isotope monitoring gave an error of only 2% (77 ng Se kg^-1^ calculated from the calibration curve for a theoretical value of 75 ng Se kg^-1^ injected) whereas quantitation based on the ^80^Se isotope monitoring gave an error of 42% (Data shown in S6 Protocol). This difference confirms that isotope 80 was affected by inorganic constituents from the biological matrix at low concentrations. For that reason we choose to select the isotope 78 for quantitation in pharmacology. The retention time of [Se-Se]-AVP peptide was observed as expected at 3.0 minutes. A second peak at 5.7 minutes was also detected from ^78^Se isotope measurement chromatogram. The fact that this signal was also recorded from all other selenium isotope monitoring indicated that this additional peak was probably issued from selenium-containing proteins present as traces in the biological matrix. The LOQ of 57 ng Se L^-1^ (57 ppt of Se) corresponds to 72 femtomoles of injected Se. Considering that the studied peptide possesses two selenium atoms, the LOQ determined on the organic content is 36 femtomoles of injected peptide. This result confirmed that the conceived analytical protocol is very robust and can be applied to biological issues.

#### Application to pharmacology: Determination of the dissociation constant (Kd) in the case of the AVP/V1A receptor system

**Fig 7:**
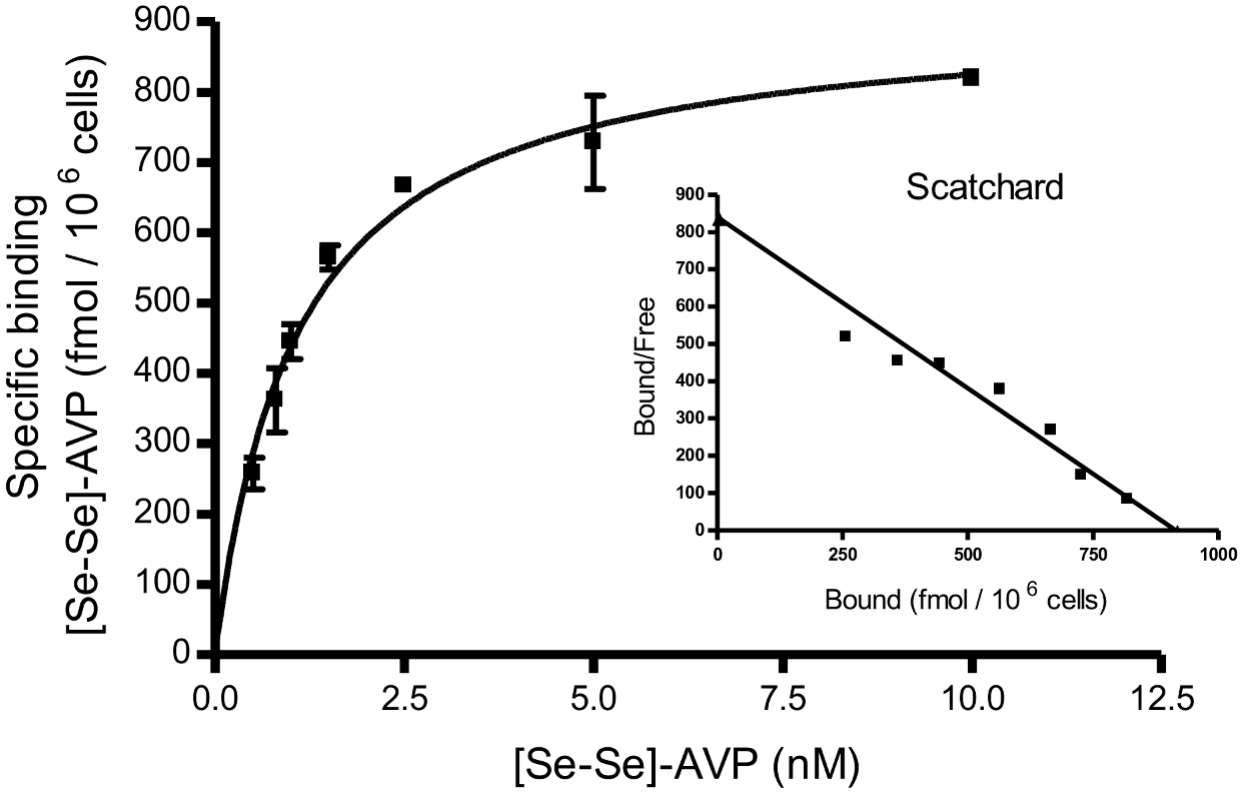
Specific binding of [Se-Se]-AVP peptide on V1A receptor expressed on CHO cells. Inset, Scatchard linear transformation of the data. Values represented on the graph are from a representative experiment performed in triplicate with corresponding SD.

CHO cells transfected to express V1A receptors were used to investigate the affinity of the prepared selenied ligand ([Se-Se]-AVP peptide). Up to now, radiolabelling of the ligand and subsequent radioactivity measurement represents state of the art methodology for such study. The results recorded with the described RP-LC-ICP-MS procedure were compared with the ones gathered with the reference method. In both analytical strategies, the dissociation constant (Kd) was deduced from 2 sets of experiments conducted to evaluate total and non-specific interactions. From these data, the specific affinity was obtained allowing the calculation of the maximum binding capacity (Bmax) necessary for Kd value determination. For such so-called saturation experiment, increasing concentrations of selenium labelled peptide were incubated on cells to determine the total binding. Similarly, [Se-Se]-AVP solutions at the same concentration levels but doped with a 2000-fold excess of native AVP were used to evaluate the unspecific binding. The equilibrium was considered being reached after 4 h of incubation at 4°C. Cells were washed to remove all unbound material. The next stage implied the recovery of the anchored ligands by incubating the cells with a dissociation solution (1% TFA in water (v/v)). After centrifugation to remove insoluble biological material, supernatant was directly analyzed by RP-LC-ICP-MS. Assays were performed in triplicate. The maximum capacity binding (Bmax) and the dissociation constant at the equilibrium (Kd) was determined with help of PRISM software. Specific curve (deduced from total and unspecific data) and Scatchard linearization from PRISM data treatment are displayed in Fig 7. Total, unspecific and specific curves are supplied in S6 Protocol. Kd of [Se-Se]-AVP on CHO cells expressing V1A receptors was 0.99±0.14 (SD) nM and Bmax was 1058±202 (SD) fmol per 10^6^ cells (mean of 2 independent experiments carried out in triplicate). The Kd value fitted well with the value determined according to conventional radiolabelling methodology serving as a reference established at 0.67±0.17 nM [31].

## Conclusion

Targeting a peptide which is present as trace concentration in complex solutions containing many salts, organic compounds and proteins necessitates a very sensitive, specific and reliable quantification methodology. Labelling the peptide of interest with selenium atoms was found to be easy to achieve according to standard solid-phase peptide synthesis. Among all mass spectrometry technologies allowing quantification, ICP-MS was found particularly appropriate provided that the two following items are satisfied. First of all, the use of a collision reaction cell in combination with the selection of the proper selenium isotope that was less hampered by polyatomic interferences was compulsory for accurate quantitation. Nevertheless, keeping just these parameters under consideration was not enough. Coupling the mass spectrometer with a liquid chromatography and optimizing the chromatographic elution was of utmost importance to discard salts and polar contaminants in neat water elution to perform relevant targeted selenium-labelled peptide concentration measurement that was successfully demonstrated in the case of pharmacological saturation experiment providing figures of merit similar to the ones recorded with conventional radiolabelling methodology. The versatility of the methodology makes it very attractive to pharmacologists and any biologists confronted with the quantitative evaluation of peptide present as trace level in complex samples.

## Supporting Information

S1 Protocol Peptide synthesis. *S1.1. Se-4-(methylbenzyl)-L-selenocysteine. S1.2. N^α^-tert-Butyloxycarbonyl-Se-4-(methylbenzyl)-L-selenocysteine. S1.3. Peptide synthesis: [Se-Se]-AVP*.
(DOC)

S2 Protocol Peptide quality control. *S2.1 MALDI-Tof analysis. S2.2. LC-ESI-MS experiment. S2.3. Net peptide Content (NPC) determination*.
(DOC)

S3 Protocol Pharmacological assays. (DOC)

S4 Protocol LC-ICP-MS method. *S4.1. Injection mode: FIA-ICP-MS vs RP-LC-ICP-MS. S4.2. Elution conditions: Organic solvent. S4.3. Elution conditions: Gradient tables. S4.4. Elution conditions: Acid formic content in mobile phase*.
(DOC)

S5 Protocol Quantification method. (DOC)

S6 Protocol Quantitation in pharmacology. *S6.1. Biological matrix spiked with [Se-Se]-AVP. S6.2. Application to pharmacology: Determination of the dissociation constant (Kd) in the case of the AVP/V1A receptor system*.
(DOC)

S1 Table (DOC)
