# Supplementary material for: Investigation of Elemental Mass Spectrometry in Pharmacology for Peptide Quantitation at Femtomolar Levels
Source: PLoS One. 2016 Jun 23;11(6):e0157943. doi: 10.1371/journal.pone.0157943 (PMC4918930; doi:10.1371/journal.pone.0157943)
Supplement: S1 Protocol — S1.1. Se-4-(methylbenzyl)-L-selenocysteine. S1.2. Nα-tert-Butyloxycarbonyl-Se-4-(methylbenzyl)-L-selenocysteine. S1.3. Peptide synthesis: [Se-Se]-AVP. (DOC) [file pone.0157943.s001.doc]

***S1 Protocol.*** *Peptide synthesis*

*S1.1. Se-4-(methylbenzyl)-L-selenocysteine*

L-selenonocystine (5.55 mmoles) was dissolved in 7.5 ml of NaOH aqueous solution (1 M). Solution was cooled in ice bath and aqueous solution of NaBH_4_ (58.3 mmoles in 7.5 ml of water) was portion wise added under argon atmosphere. Mixture was then stirred at room temperature for 30 min until became colorless. Solution was cooled again to 0°C and pH was adjusted to 7 with glacial acetic acid solution. Then 4-methylbenzyl bromide (35.8 mmoles) in ethanol (11.5 ml) was added dropwise over 30 min, and the reaction was stirred for additional 3 h under argon atmosphere at 0°C. The product was precipitated by acidification to pH 2 with HCl 6 M addition. The white precipitate was filtrated, washed with diethyl ether and dried under vacuum, 2.05 g (7.5 mmol, 68 %) were obtained.

[M+H]^+^ (^80^Se): 274.2 Da

^1^H (300 MHz, CD_3_OD + TFA) δ ppm : 7.2 (d, 2H, J_H7-H6_ = 6 Hz ) ; 7.1 (d, 2H, J_H6-H7_ = 9 Hz); 4.0 (dd, 1H, J_H4-H3_ = 6 Hz, J_H4-H3’_ = 9 Hz) ; 3.9 (s, 2H, *NH_2_*) ; 3.05 (dd, 1H, J_H3-H3’_ = 15 Hz, J_H3-H4_ = 6 Hz) ; 2.95 (dd, 1H, J_H3’-H3_ = 15 Hz, J_H3’-H4_ = 9 Hz) ; 2.3 (s, 3H, *CH_3_*).

*S1.2. N^α^-tert-Butyloxycarbonyl-Se-4-(methylbenzyl)-L-selenocysteine*

Se-4-(methylbenzyl)-L-selenocysteine (7.5 mmol) was solubilised in water (13 ml) with K_2_CO_3_ (14.07 mmol). The solution was slightly heated until complete dissolution. Then di-tert-butyl-dicarbonate (7.8 mmol) in 16 ml of 1,4-dioxane was slowly added, and the reaction mixture was stirred for 1.5 h at room temperature. 60 ml of water was then added and the solution was washed 2 times with 60 ml of diethyl ether. Aqueous layer was acidified to pH 4 with solid citric acid and then extracted 3 times with 60 ml of ethyl acetate. Organic layer was washed with a solution of 10 % citric acid, brine and dried over MgSO_4_. After evaporation under vacuum, compound was recrystallized in ether/petroleum ether to give 1.03 g (2.77 mmol, 37 %) of white powder.

[M+H]^+^ (^80^Se): 374.2 Da

^1^H RMN (600 MHz, CD_3_OD + TFA) δ ppm : 7.21 (d, 2H, J_H7-H6_ = 12 Hz ) ; 7.10 (d, 2H, J_H6-H7_ = 18 Hz); 4.35 (dd, 1H, J_H4-H3_ = 12 Hz, J_H4-H3’_ = 18 Hz) ; 3.83 (dd, 2H, J_H2-H6_ = 9 Hz ) ; 2.91 (dd, 1H, J_H3-H3’_ = 6 Hz, J_H3-H4_ = 12 Hz) ; 2.95 (dd, 1H, J_H3’-H3_ = 24 Hz, J_H3’-H4_ = 18 Hz) ; 2.31 (s, 3H, *CH_3_*), 1.47 (s, 9H, *tBu*). ^13^C RMN (600 MHz, CD_3_OD + TFA) δ ppm : 174.4 (C_9_) ; 173.4 (C_5_) ; 157.7 (C_10_) ; 137.4 (C_6_) ; 137.3 (C_6’_) ; 130.1 (C_7_) ; 129.9 (C_7’_) ; 80.7 (C_1_) ; 68.1 (C_8_) ; 55.1 (C_4_) ; 43.8 (C_2_) ; 28.7 (C_3_) ; 28.0 ; 27.6 ; 25.8 (C_11_) ; 21.14 (C_12_).

*S1.3. Peptide synthesis: [Se-Se]-AVP*

N-α Boc protected amino acids, coupling reagents: HBTU [2-(1-H-benzotriazol-1-yl)-1,1,3,3-tetra-methyluronium hexafluorophosphate] and HATU 2-(7-Aza-1H-benzotriazole-1-yl)-1,1,3,3-tetramethyluronium hexafluorophosphate and resin were purchased from Iris Biotech GmbH (Germany). N,N-dimethylformamide (DMF), methanol, acetonitrile, ethyl ether, trifluoroacetic acid (TFA), N,N-diisopropylethylamine (DIEA), triisopropylsilane (TIS) were purchased from Carlo Erba (Val de Reuil, France) or Acros organics (Noisy le Grand, France) and used without purification. Fluorhydric acid (HF) was purchased from GHC Gerling Holz&Co (Germany). Peptide was synthesized by manual solid-phase peptide synthesis (SPPS) using the Boc/Bzl strategy on a MBHA resin (loading 0.86 mmol g-1) in 0.25 mmol scale. Classic coupling step were carried out with 4 equivalents of N-protected amino-acids pre-activated using HBTU/DIEA (4 equivalents each) in DMF (2 ml mmol-1) during 15 minutes. Boc-Sec(MeBzl)-OH was introduced with 2 equivalents and activated with HATU/DIEA (2 equivalents each) and coupling time was increased to 1 h. Boc deprotection was carried out using trifluoroacetic acid (TFA) 100 % (2 x 1min). Washing steps were performed between coupling and deprotection steps with N,N-dimethylformamide. Once peptide synthesis was terminated, resin was washed with methanol and diethyl ether and then dried under vaccum and N_2_ flow. Peptide was cleaved from resin during 2 hours at 0°C in a solution of HF/p-cresol/p-thiocresol (18/1/1; v/v/v) (10 ml for 300 mg of peptide/resin). After HF evaporation the crude peptide was precipitated with cold diethyl ether. The supernatant was removed and the precipitate was dissolved in CH_3_CN/water (50/50, v/v) solution. After reverse phase purification and freeze-drying, 218.4 mg (0.18 mmol) of white powder was obtained with an overall yield of 74%.
