# Supplementary material for: Investigation of Elemental Mass Spectrometry in Pharmacology for Peptide Quantitation at Femtomolar Levels
Source: PLoS One. 2016 Jun 23;11(6):e0157943. doi: 10.1371/journal.pone.0157943 (PMC4918930; doi:10.1371/journal.pone.0157943)
Supplement: S2 Protocol — S2.1 MALDI-Tof analysis. S2.2. LC-ESI-MS experiment. S2.3. Net peptide Content (NPC) determination. (DOC) [file pone.0157943.s002.doc]

***S2 Peptide quality control***

*S2.1 MALDI-Tof analysis*

MALDI mass spectra were recorded on an Ultraflex III TOF/TOF instrument (Bruker Daltonics, Wissembourg, France) equipped with LIFT capability. A pulsed Nd:YAG laser at a wavelength of 355 nm was operated at a frequency of 100 Hz (MS data) or 200 Hz (MS/MS data) with a delayed extraction time of 30 ns. The source was operated in the positive mode. Data were acquired with the Flex Control software and processed with the Flex Analysis software. A solution of the α-cyano-4-hydroxycinnamic acid (HCCA) matrix in water/acetonitrile (70/30, v/v) at a concentration of 10 mg ml-1 was mixed with the peptide sample in equal amount and 1.2 µl of this solution was deposited onto the MALDI target according to the dried droplet procedure. After evaporation of the solvent, the MALDI target was introduced into the mass spectrometer ion source. External calibration was performed with the commercial peptide mixture (Calibration peptide standard 2, Bruker Daltonics, Wissembourg, France). MS data were acquired under the following MS conditions. An acceleration voltage of 25.0 kV (IS1) was applied for a final acceleration of 21.95 kV (IS2). The reflectron mode was used for the Tof analyzer (voltages of 26.3 kV and 13.8 kV). Mass spectra were acquired from 700 laser shots, the laser fluence being adjusted for each studied sample (Laser fluence 1). Ions were detected over a mass range from m/z 600 to 2000. MS/MS data were acquired under the following conditions. An acceleration voltage of 8.0 kV (IS1) was applied for a final acceleration of 7.25 kV (IS2). The reflectron mode was used for the Tof analyzer (voltages of 29.5 kV and 13.9 kV). Mass spectra were acquired from 700 laser shots, the laser fluence being adjusted for each studied peptide above the threshold for generation of molecular ions (Laser fluence 2 > Laser fluence 1). MS/MS experiments were performed under laser induced dissociation (LID) conditions with the LIFT cell voltage parameters set at 19.0 kV (LIFT 1) and 3.2 kV (LIFT 2) for a final acceleration of 29.5 kV (reflector voltage) and a pressure in the LIFT cell around 4 x 10^-7^ mbar. The precursor ion selector was set manually to the selenium isotope 80 peak of the molecular ion pattern for all analyses. For LID experiments, no collision gas was added (gas off spectra).

4

1

2

3

**MALDI mass spectrum of [Se-Se]-AVP (list of major molecular ions in the following table)**

| **Signal** | **m/z** | **Isotope** | **ion** |
| --- | --- | --- | --- |
| 1 | 1180.43 | ^80^Se | [M+H]^+^ of [Se-Se]-AVP |
| 2 | 1202.77 | ^80^Se | [M+Na]^+^ of [Se-Se]-AVP |
| 3 | 1052.29 | ^80^Se | [M+H]^+^ of [Se-Se]-AVP with glutamine deletion |
| 4 | 1369.44 | ^80^Se | [M+H]^+^ of [Se-Se]-AVP with double incorporation of phenylalanine and N-acetylation |

MALDI MS/MS spectrum of [M+H]^+^ ion of [Se-Se]-AVP: 1180.3 Da

(list of major fragment ion in the following table)

| **m/z (^80^Se)** | **Major fragment ions** |
| --- | --- |
| 1097.7 | loss of H_2_Se |
| 1017.9 | loss of H_2_Se_2_ |
| 850.5 | b_6_ |
| 805.07 | b_6_ – NHCONH_2_ |
| 575.6 | b(NUUY) |
| 539.5 | b(QNUU) |
| 458.7 | b(UYF) |
| 389.8 | b(FQN) |
| 372.8 | b(FQN) – NH_3_ |
| 327.9 | y_3_ |
| 310.8 | y_3_ – NH_3_ |
| 275.8 | b(FQ) |
| 242.8 | b(QN) |

*S2.2. LC-ESI-MS experiment*

LC-ESI-MS experiment was carried out on a UPLC (Acquity QSM from Waters) coupled to ESI-QTof apparatus (Synapt G2S, from Waters). Analysis was realized on a column Kinetex C18 100 x 2.1 mm, 2.6 µm, 100 Å, the applied gradient was from 0 % to 50 % of acetonitrile (with 0.1 % formic acid) in 15 min with a flow rate of 0.5 ml min-1.

**LC-UV chromatogram of [Se-Se]-AVP**

**ESI (+) mass spectrum of [Se-Se]-AVP**

*S2.3. Net peptide Content (NPC) determination*

The elemental analysis was carried out on an ElementarVario Micro Cube and compound was weighed on a Mettler Toledo UMX5Comparator balance with a precision of 0.1 µg. Nitrogen, carbon, hydrogen and suffer was simultaneously quantified. Sample was weighed in a tin capsule, first catalytic combustion of the capsule and sample was realized a 1150°C, the formed combustion gases was reduced on hot copper at 850°C and then carried with a helium flow. Gaseous mixture was separated on TDP column (desorption column with a programmable temperature; Elementar patent) and signal was detected using TCD katharometer (thermal conductivity detector).
