# Supplementary material for: Investigation of Elemental Mass Spectrometry in Pharmacology for Peptide Quantitation at Femtomolar Levels
Source: PLoS One. 2016 Jun 23;11(6):e0157943. doi: 10.1371/journal.pone.0157943 (PMC4918930; doi:10.1371/journal.pone.0157943)
Supplement: S3 Protocol — (DOC) [file pone.0157943.s003.doc]

***S3 Pharmacological protocol***

Competition assays were performed using CHO cells stably expressing the human V1A receptor. Experiments were carried out in 100 µL of binding buffer at pH 7.4 (PBS with 1mM of aqueous solution of CaCl_2_; 5 mM of aqueous solution of MgCl_2_; 0.1% of bovine serum albumin (BSA); 40 µg mL^-1^ of Bacitracine and 1 mM of PMSF), on CHO cells previously sowed on 96 multi-wells plate. The iodinated linear peptidic V1A antagonist [^125^I]OH-LVA [1, 2] was used as reference ligand and was introduced at constant concentration (0.16 nM) in presence of increasing concentrations (10^-11^ to 10^-5^ M) of competitor (AVP or [Se-Se]-AVP). Incubation was performed for 4 h at 4°C. Assays were performed in quadruplicate and in duplicate. After incubation, cells were washed three times on ice with 100 µL of binding buffer and then dissociated in 200 µL of 0.1 N NaOH. The bound radioactivity on the collected suspension was counted in a Beckman gamma counter. Data were treated with non-linear model fitting programs (GraphPad PRISM 4).

Values represented on the graph are means ± SD from 2 independent experiments performed in quadruplicate and duplicate.

[1] J. Elands, C. Barberis, S. Jard, E. Tribollet, J. Dreifuss, K. Bankowski, M. Manning, W.H. Sawyer, Eur J. Pharmacol. 147 (1987) 197-207.

[2] M. Manning, K. Bankowski, C. Barberis, S. Jard, J. Elands, W.Y. Chan Int. J. Peptide Protein Res. 40 (1992) 261-267.
