# Supplementary material for: Investigation of Elemental Mass Spectrometry in Pharmacology for Peptide Quantitation at Femtomolar Levels
Source: PLoS One. 2016 Jun 23;11(6):e0157943. doi: 10.1371/journal.pone.0157943 (PMC4918930; doi:10.1371/journal.pone.0157943)

***S4. Protocol. LC-ICP-MS method***

*S4.1. Injection mode : FIA-ICP-MS vs RP-LC-ICP-MS*

Overlaid chromatograms obtained with ^80^Se monitoring:


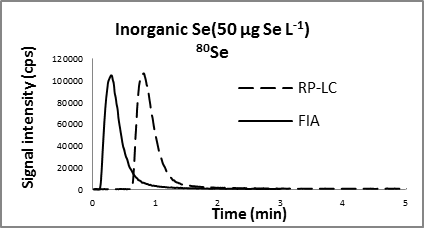

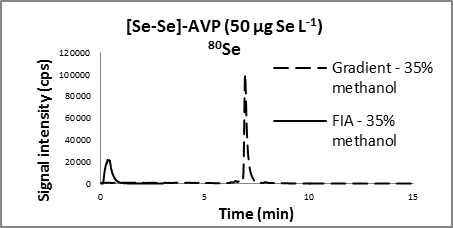


Area integration:

|  |  |  |  |  | Theoretical isotopic ratios | | |
| --- | --- | --- | --- | --- | --- | --- | --- |
| Elution with methanol |  | Area | | | 32% | 16% | 48% |
|  |  | ^77^Se | ^78^Se | ^80^Se | ^77^Se/^78^Se | ^77^Se/^80^Se | ^78^Se/^80^Se |
| Inorganic selenium 50 µg Se L^-1^ | RP-LC | 81505 | 269485 | 665540 | 30% | 12% | 40% |
|  | FIA | 73681 | 244482 | 595958 | 30% | 12% | 41% |
| [Se-Se]-AVP  50 µg Se L^-1^ | RP-LC | 36133 | 120139 | 287729 | 30% | 13% | 42% |
|  | FIA | 19803 | 65708 | 146844 | 30% | 13% | 45% |


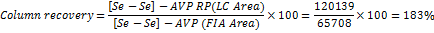


*S4.2. Elution conditions: Organic solvent*

Overlaid LC-ICP-MS chromatograms of [Se-Se]-AVP (right panel) and inorganic selenium (left panel) with methanol (CH_3_OH) and acetonitrile (CH_3_CN) elution with isotope 78 (upper panel) and isotope 80 (down panel) monitoring.


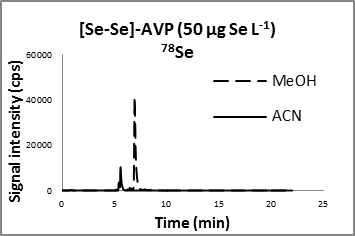

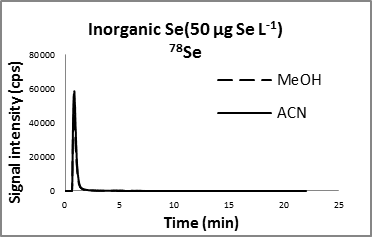


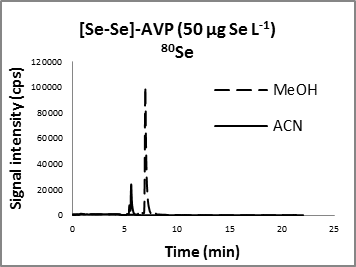

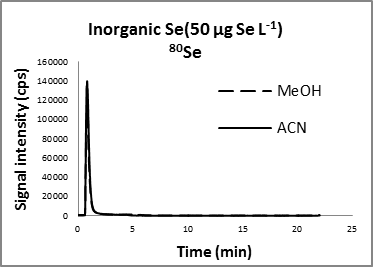


*S4.3. Elution conditions: Gradient tables*

|  | **Area** | | | **Isotopic ratio** | | |
| --- | --- | --- | --- | --- | --- | --- |
|  | ^77^Se | ^78^Se | ^80^Se | ^77^Se/^78^Se | ^77^Se/^80^Se | ^78^Se/^80^Se |
| **Gradient 11% CH_3_OH min^-1^** |  |  |  |  |  |  |
| [Se-Se]-AVP (50 ng Se L^-1^) -Injection 1 | 129 020 | 426 635 | 1 009 336 | 30% | 13% | 42% |
| [Se-Se]-AVP (50 ng Se L^-1^) -Injection 2 | 132 109 | 437 755 | 1 040 636 | 30% | 13% | 42% |
| [Se-Se]-AVP (50 ng Se L^-1^) -Injection 3 | 129 327 | 430 129 | 1 014 526 | 30% | 13% | 42% |
| Mean | **130 152** | **431 506** | **1 021 499** |  |  |  |
| RSD | 1.3% | 1.3% | 1.6% |  |  |  |
| **Isocratic 35% CH_3_OH** |  |  |  |  |  |  |
| [Se-Se]-AVP (50 ng Se L^-1^) -Injection 1 | 153 589 | 502 349 | 1 172 922 | 31% | 13% | 43% |
| [Se-Se]-AVP (50 ng Se L^-1^) -Injection 2 | 154 399 | 507 266 | 1 174 422 | 30% | 13% | 43% |
| [Se-Se]-AVP (50 ng Se L^-1^) -Injection 3 | 150 687 | 497 263 | 1 166 396 | 30% | 13% | 43% |
| Mean | **152 892** | **502 293** | **1 171 247** |  |  |  |
| RSD | 1.3% | 1.0% | 0.4% |  |  |  |
| **Isocratic 50% CH_3_OH** |  |  |  |  |  |  |
| [Se-Se]-AVP (50 ng Se L^-1^) -Injection 1 | 154 422 | 506 345 | 1 180 675 | 30% | 13% | 43% |
| [Se-Se]-AVP (50 ng Se L^-1^) -Injection 2 | 151 148 | 501 709 | 1 169 867 | 30% | 13% | 43% |
| [Se-Se]-AVP (50 ng Se L^-1^) -Injection 3 | 150 615 | 493 626 | 1 149 832 | 31% | 13% | 43% |
| Mean | **152 062** | **500 560** | **1 166 791** |  |  |  |
| RSD | 1.4% | 1.3% | 1.3% |  |  |  |
|  |  |  |  |  |  |  |
| **Gradient 33% CH_3_OH min^-1^** |  |  |  |  |  |  |
| [Se-Se]-AVP (50 ng Se L^-1^) -Injection 1 | 185 819 | 609 267 | 1 424 767 | 30% | 13% | 43% |
| [Se-Se]-AVP (50 ng Se L^-1^) -Injection 2 | 180 099 | 597 310 | 1 385 487 | 30% | 13% | 43% |
| [Se-Se]-AVP (50 ng Se L^-1^) -Injection 3 | 180 775 | 596 931 | 1 398 258 | 30% | 13% | 43% |
| Mean | **182 231** | **601 169** | **1 402 837** |  |  |  |
| RSD | 1.7% | 1.2% | 1.4% |  |  |  |

Table of areas obtained for each monitored isotopes (77, 78, 80)

*S4.4. Elution conditions: Acid formic content in mobile phase*

Overlaid LC-ICP-MS chromatograms of [Se-Se]-AVP (50 ng Se L^-1^) with different formic acid proportions


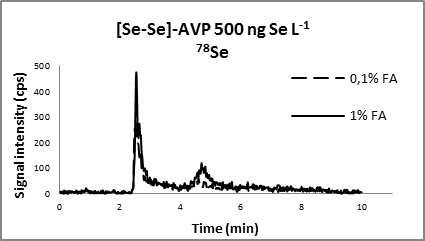

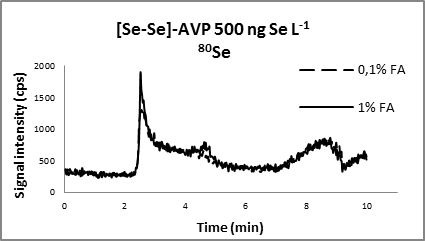

Supplement: S4 Protocol — S4.1. Injection mode: FIA-ICP-MS vs RP-LC-ICP-MS. S4.2. Elution conditions: Organic solvent. S4.3. Elution conditions: Gradient tables. S4.4. Elution conditions: Acid formic content in mobile phase. (DOC) [file pone.0157943.s004.doc]
