# Supplementary material for: Investigation of Elemental Mass Spectrometry in Pharmacology for Peptide Quantitation at Femtomolar Levels
Source: PLoS One. 2016 Jun 23;11(6):e0157943. doi: 10.1371/journal.pone.0157943 (PMC4918930; doi:10.1371/journal.pone.0157943)
Supplement: S5 Protocol — (DOC) [file pone.0157943.s005.doc]

***S5. Protocol.*** *Quantitation*

*S5.2. Calibration curve*

Recorded data with ^80^Se monitoring :

| **[Se-Se]-AVP (ng Se L^-1^)** | **[Se-Se]-AVP (nM)** | **Area ^80^Se** | **Corrected Area** |
| --- | --- | --- | --- |
| 0 | 0 | 10115 | 0 |
| 25 | 0.16 | 10819 | 704 |
| 50 | 0.32 | 11272 | 1157 |
| 75 | 0.47 | 12781 | 2666 |
| 250 | 1.58 | 17364 | 7249 |
| 500 | 3.17 | 24839 | 14724 |
| 1000 | 6.33 | 38227 | 28112 |


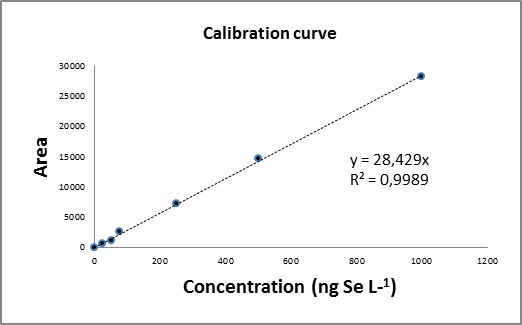


**Calibration curve with isotope 80 of selenium**

| **Injected concentration**  **(ng Se L^-1)^** | **Calculated concentration**  **from calibration curve**  **(ng Se L^-1^)** | **Bias (%)** |
| --- | --- | --- |
| **50** | 40.7 | 18.6 |
| **250** | 255.0 | 2.0 |
| **1000** | 988.8 | 1.1 |

**Method accuracy**
