# Supplementary material for: Investigation of Elemental Mass Spectrometry in Pharmacology for Peptide Quantitation at Femtomolar Levels
Source: PLoS One. 2016 Jun 23;11(6):e0157943. doi: 10.1371/journal.pone.0157943 (PMC4918930; doi:10.1371/journal.pone.0157943)
Supplement: S6 Protocol — S6.1. Biological matrix spiked with [Se-Se]-AVP. S6.2. Application to pharmacology: Determination of the dissociation constant (Kd) in the case of the AVP/V1A receptor system. (DOC) [file pone.0157943.s006.doc]

***S6. Protocol.*** *Quantification in pharmacology*

*S6.1. Biological matrix spiked with [Se-Se]-AVP*

|  | **^78^Se** | **^80^Se** |
| --- | --- | --- |
| Area [Se-Se]-AVP 75 ng Se L^-1^ | 959 | 3022 |
| Calculated concentration [Se-Se]-AVP (ng Se L^-1^) | 76.6 | 106.3 |
| Bias % | 2.1 | 41.7 |

**Bias evaluation for biological matrix spiked with [Se-Se]-AVP 75 ng Se L^-1^**

*S6.2. Application to pharmacology: Determination of the dissociation constant (Kd) in the case of the AVP/V1a receptor system.*

Total (blue), unspecific (green) and specific (red) curves from PRISM data treatment:

Saturation equilibrium of total, unspecific and specific binding of [Se-Se]-AVP peptide to CHO V1A cells. No unspecific binding was observed. Values represented on the graph are from a representative experiment performed in triplicate with corresponding SD.
