# Supplementary material for: Investigation of Elemental Mass Spectrometry in Pharmacology for Peptide Quantitation at Femtomolar Levels
Source: PLoS One. 2016 Jun 23;11(6):e0157943. doi: 10.1371/journal.pone.0157943 (PMC4918930; doi:10.1371/journal.pone.0157943)
Supplement: S1 Table — (DOC) [file pone.0157943.s007.doc]

| **Selenium stable isotope** | **^74^Se** | **^76^Se** | **^77^Se** | **^78^Se** | **^80^Se** | **^82^Se** |
| --- | --- | --- | --- | --- | --- | --- |
| **Natural abundance** | **0,89%** | **9,37%** | **7,63%** | **23,77%** | **49,61%** | **8,73%** |
| **Isobaric interferences** | ^74^Ge | ^76^Ge |  | ^78^Kr | ^80^Kr | ^82^Kr |
| **Polyatomic interferences** | ^36^Ar^38^Ar | ^38^Ar^38^Ar | ^39^K^38^Ar | ^38^Ar^40^Ar | ^40^Ar^40^Ar | ^40^Ar_2_^1^H_2_ |
|  | ^38^Ar^36^S | ^36^S^40^Ar | ^37^Cl^40^Ar | ^38^Ar^40^Ca | ^40^Ca^40^Ar | ^44^Ca^38^Ar |
|  | ^40^Ar^34^S | ^36^Ar^40^Ar | ^41^K^36^Ar | ^36^Ar^42^Ca | ^44^Ca^36^Ar | ^42^Ca^40^Ar |
|  | ^37^Cl^37^Cl | ^42^Ar^34^S | ^59^Co^18^O | ^40^Ar^37^Cl ^1^H | ^40^K^40^Ar | ^12^C^35^Cl_2_ |
|  |  | ^40^Ca^36^Ar | ^40^Ar^36^Ar^1^H | ^36^Ar^42^Ca | ^63^Cu^17^O | ^68^Zn^14^N |
|  |  | ^59^Co^17^O | ^38^Ar_2_^1^H | ^44^Ca^34^S | ^68^Zn^12^C | ^65^Cu^17^O |
|  |  | ^64^Zn^12^C | ^39^K^38^Ar | ^66^Zn^12^C |  | ^64^Zn^18^O |
|  |  | ^39^K^37^Cl | ^42^Ca^35^Cl | ^41^K^37^Cl |  | ^46^Ti^36^Ar |
|  |  | ^40^Ca^36^S | ^65^Cu^12^C | ^64^Ni^14^N |  |  |
|  |  | ^42^Ca^34^S | ^40^Ca^37^Cl | ^64^Zn^14^N |  |  |
|  |  | ^31^P_2_^14^N | ^63^Cu^14^N |  |  |  |
|  |  | ^41^K^35^Cl |  |  |  |  |
| **Polyatomic interferences (Oxydes)** |  | ^58^Ni^18^O |  | ^60^Ni^18^O | ^64^Zn^16^O^+^ | ^66^Zn^16^O^+^ |
|  |  | ^60^Ni^16^O^+^ | ^61^Ni^16^O^+^ |  | ^64^Ni^16^O^+^ |  |
| **Isobaric doubly charged ions** |  | ^152^Eu^++^ | ^154^Gd^++^ | ^156^Gd^++^ | ^160^Gd^++^ | ^164^Dy^++^ |
|  |  | ^152^Sm^++^ | ^154^Sm^++^ | ^156^Dy^++^ | ^160^Dy^++^ | ^164^Er^++^ |

**S1 Table. Possible interferences for selenium isotopes**
